# Analysis of Neurosyphilis Imaging Methods and Treatment: A Focused Review

**DOI:** 10.7759/cureus.72976

**Published:** 2024-11-04

**Authors:** Sagar S Patel, Andrew L Blum, Robert T Morgan, Brian J Piper, Angel J Rodriguez, Roger E VanVarick

**Affiliations:** 1 Medical Education, Geisinger Commonwealth School of Medicine, Scranton, USA

**Keywords:** cerebrospinal fluid, magnetic resonance imaging, neurosyphilis, penicillin g, serology testing, single photon emission tomography (spect), spect, syphilis, treponema pallidum

## Abstract

Neurosyphilis, a severe complication of syphilis caused by *Treponema pallidum*, progresses through multiple stages, including asymptomatic, meningeal, meningovascular, and late parenchymal forms such as syphilitic paresis and tabes dorsalis. Neurosyphilis spreads through sexual contact and from mother to child. Symptoms of neurosyphilis include nausea, cranial nerve deficiencies, and seizures. Diagnosing this condition is particularly challenging due to its varied symptoms, which frequently overlap with other neurological disorders. Current diagnostic approaches primarily rely on cerebrospinal fluid (CSF) analysis and neuroimaging techniques like magnetic resonance imaging (MRI) and single-photon emission computed tomography (SPECT). The exploration of recent advances in neurosyphilis testing and the expanding role of imaging techniques in tracking treatment efficacy are seen. Penicillin G continues to be the primary treatment, with ceftriaxone serving as an alternative for patients with penicillin allergies. Even though ceftriaxone is less potent than penicillin G, it can still successfully eradicate neurosyphilis in patients. This review seeks to deepen the understanding of neurosyphilis pathophysiology, refine diagnostic accuracy, and inform evidence-based treatment approaches. This will ultimately contribute to improved patient outcomes.

## Introduction and background

Syphilis is caused by the bacterium *Treponema pallidum* and has been named the great imitator due to its wide range of symptoms that mimic other diseases [1]. A study showed that *T. pallidum* promotes the apoptosis of microglia and inhibits the migration of microglia to evade clearance [2]. This pathogen can be transmitted through sexual contact or from mother to child, and once it infiltrates the body through the mucous membranes or skin, it can disseminate systemically [2]. The major risk factor for syphilis is unprotected sex, specifically in men who have sex with other men and those who have human immunodeficiency virus (HIV) [3]. In 2019, an investigation performed by the Global Burden of Disease reported that there was a worldwide prevalence of about 50 million syphilis cases, representing a 60% increase from 1990 to 2019 [4]. In 2020, the World Health Organization estimated about seven million cases, with the highest incidence in Southeast Asia, Latin America, the Caribbean, and sub-Saharan Africa [5,6]. Syphilis has become endemic and commonly observed in developing countries [7,8].

Neurosyphilis is an infection that invades the brain and spinal cord [1]. The manifestation of neurosyphilis is varied and can include personality changes, visual abnormalities, hearing loss, and seizures [9]. Within hours of infection, studies have reported that *T. palladium* is present in CSF [9-11]. This invasion has been reported in 40% of patients with primary syphilis and 23% of patients with secondary syphilis [11]. Neurosyphilis manifests in several forms: early stages include asymptomatic and meningeal involvement within the first year of infection, intermediate stages such as meningovascular syphilis typically develop between five and 12 years post-infection, and late stages, including syphilitic paresis and tables dorsalis, can appear more than a decade after the initial infection [1,11]. Before antibiotics were invented, asymptomatic neurosyphilis was seen in between 25% and 35% of patients with early syphilis and 13.5% of those with late forms of neurosyphilis [11]. Historically, neurosyphilis was identified in approximately one-third of all syphilis patients. Still, today it is more commonly associated with HIV-positive individuals, particularly those with low CD4+ counts and detectable HIV ribonucleic acid levels [12]. The incidence of neurosyphilis is disproportionately higher among Caucasian individuals compared to African Americans despite the latter having a higher overall incidence of syphilis [1]. Analyzing the interaction between syphilis and HIV is important, as both rates are slowly rising [13]. One reason for an increase in syphilis is improved HIV management, which has led to a decrease in safe sex behaviors [13]. Another reason is the emergence of macrolide-resistant strains of syphilis [13]. The prevalence of syphilis is inversely related to the effectiveness of public health initiatives, including the level of funding they receive [14]. 

Diagnosing neurosyphilis remains challenging due to the absence of standardized testing and the overlap of its symptoms with other neurodegenerative diseases, such as Alzheimer’s disease and amyotrophic lateral sclerosis [15]. Current diagnostic approaches rely on clinical findings, CSF analysis, and neuroimaging techniques, which can reveal damage in key brain regions, such as the cerebral cortex and meninges [16,17]. However, the lack of a definitive testing method complicates early detection and accurate diagnosis [1]. This focused review aims to critically analyze existing diagnostic procedures and treatment strategies for neurosyphilis, focusing on their effectiveness and reliability. By examining cross-sectional studies and comparing cognitive deficits in neurosyphilis patients to healthy controls, this review seeks to enhance the understanding of the disease’s pathophysiology, improve diagnostic accuracy, and guide evidence-based treatment approaches. Multiple studies were examined that explained the different stages and symptoms of neurosyphilis in patients. Investigations including pharmacological treatment options and diagnostic imaging techniques were clinically important. The insights gained will have substantial clinical implications, advancing the detection and management of neurosyphilis and contributing to the broader field of neurodegenerative disease research.

## Review

Cerebrospinal fluid serology

Currently, there is no universally accepted gold standard for diagnosing neurosyphilis [18]. In the absence of this gold standard, CSF testing is largely considered to be the most reliable way to test for this condition [18]. While clinical findings, symptomatology, and imaging techniques are valuable in the diagnostic process, a definitive diagnosis of neurosyphilis cannot be established without laboratory analysis of the CSF [1]. If a clinician suspects an infection of the nervous system, a lumbar puncture to collect CSF for evaluation is vital in determining the causative microbe and initiating sensitive antibiotics to cleanse the pathogen [19].

CSF investigations for neurosyphilis primarily fall into two categories: treponemal tests and nontreponemal tests [1]. Both are routinely used in tandem to increase diagnostic accuracy [1]. Treponema*-*specific tests screen for the antibodies associated with proteins located on *T. pallidum*. In the United States, five treponemal tests are approved for clinical use: particle agglutination, hemagglutination assay, micro-hemagglutination assay, enzyme/chemiluminescence immunoassays, and the fluorescent treponemal antibody absorbed (FTA-ABS) test [20]. A positive result from these tests confirms the presence of syphilis antibodies but does not indicate the stage of the infection or whether it is active or treated, highlighting a key limitation of treponemal testing [1,20].

To assess the current activity of a syphilis infection, complementary nontreponemal tests are conducted, including the Venereal Disease Research Laboratory (VDRL) test, the toluidine red unheated serum test (TRUST), and the rapid plasma reagin (RPR) test. These detect nonspecific IgG and IgM antibodies targeting lipoidal antigens released from damaged cells in the CSF, thereby indicating the current presence and activity of the disease [21]. The limitation with nontreponemal tests is a lack of specificity, meaning a positive lab result signals the presence of an infection in the CSF but does not specifically recognize it as *T. pallidum* [21]. To overcome the limitations of using either test type alone, clinicians combine treponemal and nontreponemal tests, which allows for both the detection of syphilis infection and an assessment of its current activity and stage [21]. Of these three nontreponemal tests, VDRL is the most common, followed by RPR to evaluate infection activity [22]. FTA-ABS or particle agglutination are often paired with these labs to determine the involvement of syphilis specifically [23]. 

Emerging research in CSF serology is focused on refining and expanding diagnostic techniques to improve the detection of neurosyphilis. In addition to traditional antibody testing, new biomarkers are currently being researched. Research suggests that a triggering receptor found on myelocyte-2 positively correlates with neuronal damage associated with neurosyphilis and has the potential to be utilized as a potential biomarker for the condition [24]. Additionally, a recent diagnostic performance analysis was conducted to compare if TRUST, under specific conditions and guidelines, could serve as a reliable alternative to VDRL. This experiment found that with ten microliters of antigen, TRUST displayed a 98.7% agreement of qualitative results and a 91.4% similarity in the numeric measurement of antigen present when compared with VDRL testing [25]. These findings suggest that TRUST could be a viable option for neurosyphilis screening, contributing to the ongoing optimization of CSF serologic testing. With the lack of a gold standard, CSF evaluation as a diagnostic tool for neurosyphilis is paramount in screening for the disease. However, a complete and accurate diagnosis will require neural imaging [26].

Magnetic resonance imaging

MRI utilizes the position of protons within a magnetic field to produce cross-sectional slices of the body, and most importantly, allows for the visualization of internal soft tissue structures [27]. Contrary to other imaging techniques, such as SPECT, MRIs can produce images without exposing patients to radiation and therefore are popular for a wide variety of uses including diagnoses of neurological diseases [28]. Overall, MRI provides high-detail three-dimensional images that promote its use in understanding the brain's structure and inferring the probable functionality of identified structures [29]. However, a major limitation of using MRIs is that the findings can be generalized, often lacking specificity in correlating structural changes directly to specific neurological conditions [1]. In many neurological diseases, the structural changes within brain areas are a result of defects within that tissue's function, so functionality imaging techniques, such as SPECT, may provide earlier disease diagnoses [30]. Another potential limitation of MRI scanning is that the interpretation of the scan can be influenced by motion artifacts if there is movement by the patient during the scanning process. This can especially become an issue for identifying neurological diseases, such as Parkinson's disease, that cause tremors [31].

A case report of a 52-year-old man presenting with dysesthesia, neck stiffness, and headaches was examined with an MRI [32]. The results showed bilateral subcortical temporal hyperintensity and loss in cerebral parenchyma within the patient [32]. The findings of the MRI led to a suspicion of neurosyphilis, and blood serum tests confirmed *T. pallidum* with subsequent diagnosis of neurosyphilis [32]. Three-week treatment with penicillin improved the patient’s symptoms [32]. In certain cases, MRI scanning can prove useful for identifying potential neurosyphilis symptoms leading to a correct diagnosis and quick effective treatment before any progression of the disease.

A case report of a 49-year-old man with no relevant past medical history presenting with behavioral changes, memory loss, confusion, and hallucinations was examined with an MRI scan [33]. The MRI showed abnormalities in the patient’s temporal and insular lobes [33]. In conjunction with a CSF exam, the MRI results led to suspected herpes simplex virus (HSV) induced encephalitis [33]. The patient was treated with acyclovir but did not show improvements in his symptoms. The following lab exam of the patient's CSF came back negative for HSV but was positive for *T. pallidum*, indicating neurosyphilis as the cause. Following treatment with penicillin, the patient’s symptoms improved [33].

A different case report of a previously healthy 29-year-old man presenting with mental deficiencies, seizures, and motor dysphasia was examined with two MRI scans with no abnormalities detected [34]. Tests for HSV and immunodeficiency were also negative. Due to the observed symptoms, encephalitis was the initial diagnosis, and the patient received acyclovir [34]. After no improvement in symptoms, limbic encephalitis was suspected, and the patient received intravenous (IV) immunoglobin for five days. Laboratory serum tests were received and showed positive for *T. pallidum*, indicating neurosyphilis as the problem [34]. Treatment with penicillin improved his condition [34]. As seen in these case reports, neurosyphilis’ varied symptomology makes diagnosis especially difficult when relying solely on MRI scanning. Often, other diagnostic and imaging techniques are required to correctly identify neurosyphilis as the cause of observed abnormalities over other neurological conditions [35]. 

Single-photon emission computed tomography

Single-photon emission computed tomography (SPECT) is a nuclear imaging procedure used in the medical field that produces a three-dimensional image of a radioactive tracer, called a probe [36]. The probe is injected into the patient’s bloodstream and taken up by specific tissues [36]. Using specialized nuclear medicine cameras, SPECT can help clinicians assess blood perfusion and functionality of desired tissues because abnormal blood perfusion rates in sections of the body are correlated with disruptions in the metabolism of cells surrounding those blood vessels [36-38]. The ability to analyze the tissue's functionality and physiology makes SPECT imaging unique from other imaging techniques [36,39].

SPECT has proven highly effective in various clinical applications, such as evaluating brain scans in patients diagnosed with dementia, detecting cerebrovascular disease, and mapping cerebral blood flow during surgery [36,40]. Despite its effectiveness, clinicians must consider the potential risks associated with SPECT, particularly due to radiation exposure, which is of special concern in pregnant women [36,39]. Teenagers can also get syphilis; however, they can undergo SPECT imaging since the radiation usage is limited [36]. Additionally, certain patient conditions, such as obesity, may preclude the use of SPECT due to the weight limitations of the scanning equipment, as the limit is 300 pounds [36,39]. Other considerations include avoiding substances like caffeine and phosphodiesterase-3 inhibitors before the scan, as these interfere with the results [36,40]. In rare instances, patients may experience allergic reactions to the tracer compound used in the procedure [36,41].

In a particular study, the scientists utilized a cross-sectional design, as four HIV-negative neurosyphilis patients and healthy controls underwent SPECT imaging [42]. The neurosyphilis diagnosis in this analysis was based on a white blood cell (WBC) count of five cells/microliter, CSF protein levels of 45 mg/dL or more, and a positive CSF-VDRL test [42]. Brain MRIs and SPECT scans were performed numerous times. The investigation included eight male participants with an average age of 46 years [42]. The experimental group showed reduced blood flow in the frontal and posterior regions of the brain compared to the healthy controls [42]. The pattern seen from this could be associated with the cognitive decline seen in neurosyphilis patients. The findings align with previous SPECT studies that have documented reduced regional cerebral blood flow (rCBF) in the frontal lobes of patients with general paresis, a common manifestation of neurosyphilis [42,43]. The investigation did report less rCBF in the temporal region, which does show inconsistency regarding neurosyphilis patients [42]. This attributes the inconsistencies to the stage of diagnosis as the increased blood flow could show inflammatory changes in the early phase and decreased blood flow could show neuronal death [42,43]. Additionally, the report noted that reduced blood flow was seen in the posterior cingulate gyrus for these patients [42]. However, this is a limitation as a diagnostic parameter for neurosyphilis as this condition is also seen in patients who have Alzheimer’s disease [44]. Regardless of the acknowledged limitations seen within the study, SPECT imaging proved to be a valuable method for observing the patients’ progression following treatment with antibiotics as the researchers were able to monitor improvements in rCBF throughout the recovery process.

Treatment options in neurosyphilis

Based on the pharmacokinetics of existing medications, their impact on *T. pallidum* in vitro, laboratory considerations, biological plausibility, expert opinions, and clinical experience, penicillin G is considered the first-line therapy for neurosyphilis [18]. Parenteral aqueous penicillin G is the predominant treatment for neurosyphilis and is advised for all phases of illness according to the Centers for Disease Control and Prevention (CDC) guidelines [18]. Treatment for syphilis with neurologic damage is more extensive [45]. Penicillin has an adequate degree of CSF penetration, particularly at larger doses [45]. When treating neurosyphilis, aqueous penicillin G is favored over other forms of the antibiotic, including benzathine penicillin since it can reach greater CSF concentrations [45]. Penicillin primarily inhibits the development of the bacterium, weakens it, and enables the breakdown of the existing cell wall by permanently blocking the crucial enzyme required for peptidoglycan cross-linking in the bacterial cell wall [45]. When excessive water rapidly enters through osmotic pressure and causes explosive cellular lysis, the bacteria are unable to grow or mend their cell walls, which prevents them from reproducing and ultimately leads to their death [46]. A bacterial cell exposed to penicillin may also suffer oxidative damage. Aqueous crystalline penicillin G can be taken as a conventional dosage of three to four million units intravenously every four hours for 10 to 14 days or as a continuous IV infusion of 24 million units per day for the same duration [46].

As an alternate treatment, ceftriaxone is advised since it effectively crosses the blood-brain barrier and is bactericidal for *T. pallidum* at low doses [47]. In very allergic, high-risk patients, ceftriaxone skin testing can be performed to determine safety as there is minimal allergic cross-reactivity with the antibiotic [48]. According to several recent studies, ceftriaxone is as successful as the usual IV aqueous penicillin G regimen, in correcting CSF anomalies and improving clinical symptoms in neurosyphilis patients [47-49]. According to the CDC, the recommended dosage for ceftriaxone is two grams of IV daily for 10 to 14 days [48]. One gram of ceftriaxone daily for 14 days results in considerable reductions in serum and cerebrospinal fluid IgG reactivity, according to two case studies [49]. Retrospective multicenter research also found that two grams of ceftriaxone for over a week is a more efficient option than aqueous crystalline penicillin IV three to four milligrams every four to six hours for 10 days [50]. Although ceftriaxone may not be as potent as penicillin G, studies show that it can reach levels in the fluid to combat *T. pallidum* [49,50]. This provides a viable alternative treatment option effectively [50].

Impact of treatment on brain imaging

When it comes to treating neurosyphilis using penicillin G as the main treatment method, this has a noticeable impact on brain imaging outcomes [51]. Penicillin G can cross the blood-brain barrier, which helps to decrease inflammation and damage caused by the infection [45]. This improvement can be seen in follow-up brain scans like computed tomography, MRI, and brain SPECT imaging showing a decrease in abnormalities in the brain [46]. The resolution images conclude that the treatment of penicillin G is effective toward abnormal brain regions and observe the progression of *T. pallidum* being eradicated [51]. The alternative beta-lactam treatment option, ceftriaxone, does influence brain imaging results; however, the impacts of the two differ due to the efficacy of each antibiotic [50]. Ceftriaxone can achieve therapeutic concentrations in the CSF, yet its ability to resolve brain irregularities is not as distinct as penicillin G [52]. The antibiotic does display improvements by reducing the inflammation and lesions in the abnormal brain region [52]. With ceftriaxone, the recovery is less complete than penicillin G due to being less effective at eliminating the pathogen, allowing for the possibility of abnormalities to linger [52]. Physicians recommend frequent follow-up imaging to observe the treatment’s efficacy and make any adjustments depending on the severity [50].

Evaluating the optimal first-line therapy

When looking at how penicillin G and ceftriaxone affect brain scans, it is frequently observed that penicillin G tends to provide a significant resolution of abnormalities due to neurosyphilis [53]. Current research typically indicates that individuals treated with penicillin G are more likely than those getting ceftriaxone to exhibit improvements in brain alterations, such as a reduction in lesions and cortical atrophy [54]. However, this therapy has negative consequences that include prolonged hospital stays, hypersensitivity responses, and the immunologic reaction known as a Jarisch-Herxheimer reaction, which is transient [55]. The common symptoms of this reaction include fever, headache, and myalgia [55]. This occurs 24 hours after starting the treatment for syphilis [55]. Ceftriaxone is well tolerated, permeates the central nervous system (CNS), and has a half-life of seven hours [55]. This makes it appropriate for once daily dosage and has demonstrated antitreponemal behavior [55]. Numerous studies have demonstrated its efficacy in treating neurosyphilis as well as primary and secondary syphilis in certain groups, including those who are HIV-positive [55-57]. From a clinical standpoint, the short time between ACPG treatments and lengthy hospital stays add to the patient care load, which is why many patients select the ceftriaxone regimen [56]. However, there are not enough clinical studies to compare the effectiveness of these two medications to conclusively state, which can reduce more brain abnormalities [57]. According to treatment recommendations, IV penicillin G is currently the gold standard therapy for neurosyphilis [45]. Yet, both therapy regimens can enhance brain outcomes with strong blood-brain barrier penetration abilities, highlighting the need for randomized controlled trials of imaging evaluations to monitor development, and guide additional therapeutic approaches [55].

Discussion 

Asymptomatic neurosyphilis is the most common early manifestation of the disease, occurring in approximately one-third of affected patients [1,13]. Despite the absence of overt neurological symptoms, asymptomatic neurosyphilis is often diagnosed through a positive VDRL test on CSF [1,56]. Studies suggest that the presence of CSF abnormalities, such as elevated WBC counts between five and 100 cells/microliter or protein levels ranging from 45 to 100 mg/dL, increases the likelihood of developing symptomatic neurosyphilis later [1,11,57]. The second stage of early neurosyphilis is meningeal neurosyphilis, characterized by inflammation affecting the meninges and vasculature with cranial nerves VII, VIII, VI, and II affected in that order [1,58]. The spinal cord is also affected, leading to sensory loss, back pain, and leg weakness [1,59]. Syphilitic gummas, which are granulomatous lesions made up of necrotic tissue, most commonly develop in the liver and meninges, but can also occur in the skin and brain [1,60,61]. CSF analysis in this stage typically reveals more pronounced abnormalities, with WBC counts between 200 and 400 cells/microliter and protein levels ranging from 100 to 200 mg/dL [1].

The intermediate stage is called meningovascular neurosyphilis. This stage becomes prevalent about seven years after initial diagnosis [1,62]. This is when inflammation in the meninges leads to strokes and cerebrovascular problems [1]. This stage involves perivascular inflammation, which leads to thickening from the enhanced activity of fibroblasts and inflammatory changes [1,63]. The most prevalent symptom in this stage is strokes at an early age [1,64]. The vessel that is most likely affected is the middle cerebral artery [1,65]. However, any subarachnoid vessel can be affected [1,66]. Other symptoms may include vertigo, dizziness, and insomnia [1,67]. In previous studies, these symptoms are seen to be present due to inflammation of the meninges [1,68]. The spinal cord could also be affected, which leads to lower back problems, loss of the senses, and shriveling of muscles [1,69]. When examining the CSF, the WBCs/microliter are at 10 to 100, and protein levels are at 100 to 200 mg/dL [1,70].

The first part of the late stage, also called parenchymal neurosyphilis, is called syphilitic paresis. This is when patients who have dementia undergo paralysis. This is usually presented 15 to 20 years after the initial infection of neurosyphilis [1,71]. The frontal and temporal lobes are infected. This occurs when chronic meningoencephalitis results in cerebral atrophy [1,72]. Meningeal fibrosis is created, which are plaques in the frontal and parietal cortices [1,73]. This leads to loss of cerebral functioning. The healthcare provider may see a variety of symptoms, which include depression, agitation, and dysarthria [1,60]. This is usually seen in men living in developing countries and will affect about 7% of patients who are living with untreated syphilis [1,60]. When examining a patient with syphilitic paresis using magnetic resonance imaging (MRI), could show enhanced usage of the amygdala, hippocampus, and cerebral atrophy [1,61]. When analyzing CSF testing, there are 25 to 75 WBCs/L and protein levels between 50 and 100 mg/dL [1]. Treating this can be done using antibiotics [1,74]. The second part of the late stage is called tabes dorsalis. This occurs when the dorsal column and roots of the spinal cord disintegrate [1]. When this occurs, cytokines get released, reactive oxidative agents form and destroy cells, and neurons get damaged via *T. pallidum* invasion [1,62]. Patients who have this have balance issues and an uncontrollable bladder. Neurological deficits include Argyll Robertson pupils and diminished reflexes [1]. A loss of proprioception is present in this stage [1,62]. When examining a patient in this stage via MRI, this may show disintegration of the spinal cord [1,63]. Slightly elevated WBCs/microliter are seen, and protein levels are between 45 and 75 mg/dL [1].

In analyzing the extent to which neurosyphilis has impacted the brain, researchers look at diagnostic tests such as SPECT imaging, CSF parameters, and MRIs. CSF and serum laboratory tests are the primary diagnostic methods for identifying neurosyphilis, focusing on detecting elevated CSF white blood cell counts and protein levels [3,75]. These laboratory tests serve as the initial step in diagnosis, with neuroimaging techniques subsequently providing valuable insights into the extent of the infection.

Research has demonstrated the value of SPECT imaging in evaluating rCBF among neurosyphilis patients. For instance, a case report involving a 39-year-old man with confirmed neurosyphilis demonstrated multifocal hypoperfusion in the cerebral cortex via SPECT imaging, while MRI scans revealed elevated signal intensity on T2-weighted and fluid-attenuated inversion recovery images [65]. This report also investigated the thalamic-cortical circuitry, which plays a critical role in cortical excitability, establishing a potential link between the observed hypoperfusion and functional impairments [65]. Reduced rCBF in various brain regions may contribute to the dysfunction of these areas, highlighting the importance of imaging in understanding the effects of neurosyphilis on brain function. The clinical significance of these findings is attributed to diagnosing the different stages of neurosyphilis. Early stages of neurosyphilis can be distinguished from asymptomatic stages based upon hypoperfusion witnessed in regions such as the middle frontal gyrus, anterior insula, posterior cingulate gyrus, and central operculum [42].

Current research suggests that there is a positive correlation between patients who tested positive for neurosyphilis and cognitive impairment [42]. A cohort study looking at this correlation focused on eight male patients, out of which four were neurosyphilis positive [42]. These patients were assessed based on the Mini-Mental State Examination (MMSE), Clinical Dementia Rating (CDR), and Global Deterioration Scale (GDS). The MMSE, which consists of 30 questions with a top score of 30, showed that three out of four neurosyphilis-positive patients scored 25 or lower, indicating cognitive impairment [66]. The CDR, which rates dementia severity from zero (no dementia) to three (severe dementia), revealed that one patient had very mild dementia (score of 0.5), while the other three patients had mild dementia (score of one) [42,67]. GDS is based on a scale from one having no cognitive decline to seven showing very severe cognitive decline [68]. Patients one to four displayed GDS results ranging from three to five indicating mild (score of three) to moderately severe decline (score of five) [68]. These findings suggest a probable association between neurosyphilis and cognitive impairment. The clinical significance of these tests determining a link can prove vital in the approach providers take when trying to prove neurosyphilis is present. The limitations of these tests are that if other diseases or injuries are present, then they may contribute to the observed cognitive impairment. Future studies designed to solidify this correlation are an important area of concentration.

MRI utilization has proven to be a crucial tool in diagnosing neurosyphilis. A report emphasizing the importance of MRI involvement focused on a 35-year-old man suspected of having neurosyphilis but did not meet the typical CSF diagnostic criteria [69]. However, subsequent MRI scans revealed elevated signal intensity in the bilateral mesial temporal lobes, particularly affecting the hippocampus and amygdala [69]. There have been other studies discussing a possible connection between the mesial temporal and neurosyphilis [7]. Proper interpretation of MRI scans, using both radiological and neurological views, is crucial for accurately identifying such impacts. A radiological view confirms that the patient's right side is on the left side of the image [69,70]. A neurological view ensures that the patient's right side is on the right side of the image [69,70]. The radiologic findings of this report make it plausible that the mesial temporal lobes and other regions of the cerebrum are negatively impacted by neurosyphilis infection. MRI usage is clinically significant because the CSF parameters do not always tell the whole story. This investigation reveals the limitations that are present when only looking at CSF biomarkers regarding neurosyphilis. When diagnosing, it is paramount that healthcare professionals consider many different vantage points when pinpointing exactly what the patient is suffering from. The combination of serological and radiological testing reduces the likelihood of misdiagnosis that can be witnessed if only one or the other is performed.

Neurosyphilis can be distinguished from other forms of syphilis by different biomarkers present in the CSF. A cross-sectional study analyzing these differences was performed on 59 patients with neurosyphilis and 30 patients with latent syphilis infection [73]. The study focused on various CSF parameters, including CSF-nucleated cells, CSF-TRUST, CSF-total protein, and CSF-IgG [73]. Results indicated that elevated levels of these markers are associated with blood-brain barrier dysfunction and are strongly linked to neurosyphilis [73,74]. These findings emphasize the importance of accurately interpreting serological test results to differentiate neurosyphilis from other forms of syphilis, ultimately improving diagnostic precision. The clinical significance of determining the baseline requirements for neurosyphilis to be a disease of concern is invaluable. The serological test baselines for neurosyphilis tell physicians what they should be looking for, while neuroimaging procedures tell them where they should be looking.

Penicillin is the most popular treatment for neurosyphilis. There have been studies suggesting ceftriaxone as being more effective than penicillin, however, there are few large, randomized controls to support this [51]. Studies have proven that oral amoxicillin with probenecid increases the levels of the drug in the CSF for the treatment of neurosyphilis [51]. Medications like these should be considered in future research for the positive patient outcomes they can potentially provide. Focusing on specific research groups such as pregnant women, people living with HIV, and those with penicillin allergies can lead to more desirable clinical results for treatment options [51].

Targeting the early detection of neurosyphilis can also aid in the prevention of disease progression. Regular checkups with a health care provider and routine blood work are crucial. The CDC recommends serum nontreponemal testing, which eliminates the need for repeated CSF examinations [16]. In poorer populations and those wishing to maintain privacy, there are at-home self-tests that can be effective in early detection that can then be followed up by a visit with a health care provider [76]. Vaccinations against the immunogens for *T. pallidum* are also something that is being worked on as a preventative measure for syphilis [77].

## Conclusions

This focused review underscores the importance of combining multiple diagnostic modalities to accurately diagnose and manage neurosyphilis. While each tool has its limitations, their combined use offers a powerful means of understanding the complex effects of neurosyphilis, ultimately leading to better patient care and outcomes. Future studies looking at alternative treatments for neurosyphilis regarding medicinal intervention are an area of importance. 

Unresolved *T. pallidum* infection spreads into the CNS and causes neurological abnormalities that can present as personality changes, loss of sensation, or seizures. Modern detection strategies involve a combination of CSF testing and neuroimaging, such as MRI or SPECT scans. CSF serology testing is used to determine the presence of Treponema antibodies and if there is a present infection. MRIs are the most common and safest imaging technique. SPECT imaging utilizes a radioactive probe and, in addition to searching for tissue damage and structural malfunction, these scans determine changes in perfusion to cortical regions and surrounding tissue. Although there is no clear structural pattern to the neurological damage caused by a neurosyphilis infection, studies show interest in the hippocampus, amygdala, thalamus, and several cortical regions. If a neurosyphilis infection is confirmed, the standard treatment is IV penicillin G administered regularly for approximately 10 to 14 days, as this antibiotic readily traverses the blood-brain barrier. If allergic to penicillin, ceftriaxone is a viable alternative for the treatment of neurosyphilis. MRI and SPECT scans are essential to monitor patient progress during and post-antibiotic treatment. The scanning techniques can be implemented post-treatment to assess changes in tissue function, structure, and blood flow to determine if the infection has subsided and healthier neurological function is returning. Future directions for neurosyphilis research focus on discovering accessible biomarkers that can serve as an acceptable gold standard in future diagnosis of this condition.
